# Cooperative Scheduling of Imaging Observation Tasks for High-Altitude Airships Based on Propagation Algorithm

**DOI:** 10.1100/2012/548250

**Published:** 2012-12-16

**Authors:** He Chuan, Qiu Dishan, Liu Jin

**Affiliations:** Science and Technology on Information Systems Engineering Laboratory, National University of Defense Technology, Changsha 410073, China

## Abstract

The cooperative scheduling problem on high-altitude airships for imaging observation tasks is discussed. A constraint programming model is established by analyzing the main constraints, which takes the maximum task benefit and the minimum cruising distance as two optimization objectives. The cooperative scheduling problem of high-altitude airships is converted into a main problem and a subproblem by adopting hierarchy architecture. The solution to the main problem can construct the preliminary matching between tasks and observation resource in order to reduce the search space of the original problem. Furthermore, the solution to the sub-problem can detect the key nodes that each airship needs to fly through in sequence, so as to get the cruising path. Firstly, the task set is divided by using k-core neighborhood growth cluster algorithm (K-NGCA). Then, a novel swarm intelligence algorithm named propagation algorithm (PA) is combined with the key node search algorithm (KNSA) to optimize the cruising path of each airship and determine the execution time interval of each task. Meanwhile, this paper also provides the realization approach of the above algorithm and especially makes a detailed introduction on the encoding rules, search models, and propagation mechanism of the PA. Finally, the application results and comparison analysis show the proposed models and algorithms are effective and feasible.

## 1. Introduction

The high-altitude airship is a stay-in-air aerostat, which is lifted off by static buoyancy and moved forward by propulsion device. It is normally powered by regenerative energies, such as solar cells or regenerative fuel cells. The high-altitude airship can stay 20*∼*100 km above the ground in the near space for a long time. This airspace belongs to neither the aviation field nor spaceflight field, but it has a broad prospect in many military applications. Once equipped with various payloads, it can be used to fulfill different missions, such as information acquisition, communication security, and battlefield transportation [1–4].

Nowadays, high-altitude airship located in the near space is attracting more and more interests worldwide. It is well known that some projects of high-altitude airship have been launched, for example, the Sky Tower [5] and Dark Sky Station [6] projects in USA, the Cargo Lifter [7] project in the European Union, the ETRI [8] project in Korea, and the Sky Net project in Japan [9]. China began its own high-altitude airship projects in 2002 and has already completed the design, manufacture and test flight of the low-altitude craft [10–12]. The verification airship has accomplished its low-altitude flight experiment in 2003. It is expected that the operational height of the final product is 20*∼*22 km off ground, the payload can reach 1.8 t, and the duration would be more than one year. 

As a new application platform, the high-altitude airship has many advantages compared with the imaging satellite [13–15] and conventional heavier-than-air (HTA) [16, 17] aircraft (e.g., unmanned aircraft vehicle). For instance, it has a longer endurance, a rapider response, a higher effectiveness-cost ratio, a more suitable operational altitude, and a higher viability [18–21].

**Longer Endurance**

Due to its specific flying mechanism, the high-altitude airship can successively work for several months or even longer. On the other hand, the working period of satellite is restrained by its orbit, and that of UAV is constrained by the fuel capacity.

**Rapider Response**

The lifting-off speed of the high-altitude airship can be up to 300 m/min. With such a fast lifting-off speed, it only requires 2 hours for the airship to get to the near space. In comparison, launching a new satellite requires about 40 days of preparations. Although UAV is a fast deployment aircraft, it also requires special auxiliary equipment for launching.

**Higher Effectiveness-Cost Ratio**

The high-altitude airship generally has a simpler structure and a lower cost in deployment, operation, and control. Furthermore, the high-altitude airship has a larger payload capacity than both satellite and UAV.

**More Suitable Operational Altitude**

The operational altitude of the high-altitude airship is higher than UAV but lower than the imaging satellite. Since stratosphere is below the ionosphere, the electromagnetic signal of airship would not be interfered by the ionized particles in the ionosphere.

**Higher Viability**

Airship has a naturally stealth ability for its nonmetal makeup. Both the electromagnetic and heat reflective areas of an airship are small. Moreover, the working altitude of the high-altitude airship is unlikely to be reached by normal antiaircraft weapons.

Those above features have turned high-altitude airship into an ideal imaging observation platform. In return, they also lead to many differences in control technologies (e.g., attitude control, driving control, pressure control, and position control) of high-altitude airships compared with imaging satellite and UAV. For example, altitude adjustments of airship require specific control technologies by capsule inflation and deflation.

Existing studies on high-altitude airship are scattered over a range of journals, conferences, books, and reports. Chen et al. [22] investigated the inertia propulsion system of high-altitude airship and established hydrodynamic models with three factors, that is, the velocity of the wind, the diameters, and the working altitude. Ma and Sun [23] studied the feasibility and technical difficulty of the Multipower system in high-altitude airship. Then, a new management method of the Multipower system is proposed based on the various flight modes. Rao et al. [24] focused on the mission path-following controller for the airship by applying artificial neural network (ANN). Bessert and Frederich [25] investigated the aerodynamic influence of high-altitude airship and presented a novel method to test the aerodynamics on the structural behavior of airship. The aforementioned researches mainly focused on the single high-altitude airship platform, which included cruising path control, energy system design, communication links optimization, and dynamics modeling. These researches were utilized to improve the performance of platform (e.g., reducing energy consumption, decreasing the cruising distance, and easing control). However, few studies have paid attention to the task planning, which is one of the most demanding issues for airship applications.

There are also numerous distinctions in task scheduling between high-altitude airship and other platforms. Compared with imaging satellite, high-altitude airship can cruise independently above the target for fixed-point observation because of its slow speed and suspension ability. The task execution sequence of airship is completely determined by its path choosing. Thus, the task planning for high-altitude airship is free from orbit issues faced by imaging satellite. Moreover, the high moving speed of satellite enables it with multiple chances to observe the same target during the whole task period. In contrast, restrained by the low cruising speed, high-altitude airship's revisit cycle during the task period is longer. This is also a problem needing to be noticed during task planning for high-altitude airship. Different from UAV, high-altitude airship has high mobility as well as suspension observation ability; that is, high-altitude airship is able to continuously monitor a target by long time hovering. Although UAV is also able to conduct continuous monitoring over a fixed-point target, its continuous observations are actually comprised by several dispersed investigation activities restrained by the mobility and the cruising distance. Besides, UAV usually needs to take off and land in the base, which is the starting point and the ending point of the cruising path. On the contrary, the high-altitude airship is not restricted by this factor due to its long endurance.

In this paper, we drive into the cooperative scheduling problem of high-altitude airships toward the imaging observation tasks and discuss the optimization methods of task assigning and cruising path choosing. Multiairship scheduling is a combination optimization problem that is more complex than the single airship scheduling due to the fact that the former is required to optimize the task execution schemes in each airship simultaneously. Thus, a hierarchical scheduling framework is constructed to facilitate the problem solving process. The simulation results show the effectiveness of this strategy.

The remainder of this paper is organized as follows.  Section 2 describes the collaborative programming problem of high-altitude airships and establishes the constraint programming model.  Section 3 designs the collaborative scheduling frame and proposes the solution algorithms. Simulation results and performance analysis are given in Section 4. Finally, Section 5 concludes the paper with some discussion about future research.

## 2. Problem Description and Modeling

**Figure 1:**
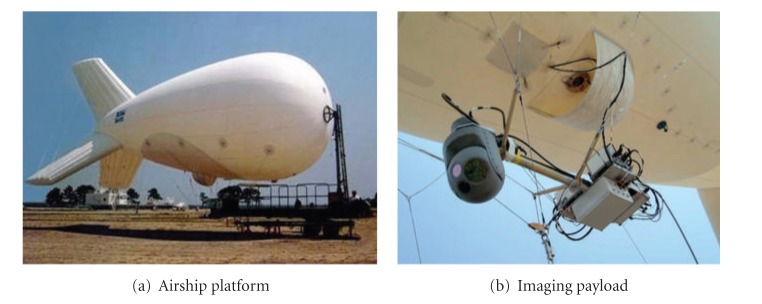
RAID system of U.S troop.

The application of the high-altitude airship into the earth imaging reconnaissance activity is an important supplement of other reconnaissance methods. The imaging payload of a high-altitude airship is usually installed in the task capsule and can swim or rotate within a certain angular range in order to observe the ground target.  Figure 1 is the reconnaissance airship which is called RAID system and used by US troops in Iraq and Afghanistan. RAID began to be equipped in 2003, and more than 60 sets of this system have been in service currently [26].

In the execution of the imaging reconnaissance tasks, the high-altitude airship maneuvers over the target area along with the cruising path and then begins the continuous observing in the hover-and-stare way. Usually, the imaging task has timeliness [27, 28] that is, each task has its time slot which is to reflect the requirements on the execution timing interval. The task execution must be finished within its time window. Otherwise, the task will lose its executive value or become invalid. Generally, imaging reconnaissance tasks are numerous and widely distributed in the battlefield. Hence, the high-altitude airship observes targets in different positions through multiple timescruising. However, due to the limitations of the cruising speed, the airship has to consume a lot of time in the cursing road. This may lead to parts of tasks that cannot be executed within their deadline. In order to maximize the overall observation benefit, it is essential to choose the observable tasks and determine the execution time interval for each airship based on the heterogeneity.

To facilitate description of this problem, we summarize the main notations which are used throughout this paper as follows:

 
*T*_*p*_ = [*t*_start_, *t*_end_] is the task period of the airship; *t*_start_ denotes the starting time of the observation activity and *t*_end_ denotes the ending time of the observation activity;
 Task = {task_1_, task_2_,…, task_*n*_} is the set of the imaging tasks, where task_*i*_ denotes the *i*th task and *n* denotes the number of the tasks;
 
*A* = {*a*_1_, *a*_2_,…, *a*_*m*_} is the set of the observation resources, where *a*_*k*_ denotes the *k*th airship and *m* denotes the number of the airships;
 
*o*_*k*_ = (*ox*_*k*_, *oy*_*k*_) is the projective coordinates of *a*_*k*_ on the ground, where *ox*_*k*_ and *oy*_*k*_ denote the horizontal coordinate and vertical coordinate, respectively;
 speed_*k*_ is the average cursing speed of *a*_*k*_;
 RP_*k*_ is The resolution of the payload attached with *a*_*k*_;
 sit_*i*_ = (*tx*_*i*_, *ty*_*i*_) is the position coordinates of task_*i*_, where *tx*_*i*_ and *ty*_*i*_ denote the horizontal coordinate and vertical coordinate, respectively;
 [*ta*_*i*_, *td*_*i*_] is the time window of task_*i*_, where *ta*_*i*_ denotes the allowable execution timing instant and *td*_*i*_ denotes the deadline;
 
*tb*_*i*_ is the beginning execution timing instant of task_*i*_;
 
*te*_*i*_ is the ending execution timing instant of task_*i*_;
 
*t*_*i*_ is the duration time (or called the task length) of task_*i*_;
 
*P*_*i*_ is the value index of task_*i*_;
 
*u*_*i*_ is the imaging resolution which needs to be satisfied in the execution of task_*i*_;
 
*D* = [*d*_*i*,*j*_]_*n*×*n*_is The distance matrix, where *d*_*i*,*j*_ denotes the distance from task_*i*_ to task_*j*_.

Due to the low-speed maneuverability and hover ability, the high-altitude airship is not restricted by the turning angle, climbing angle, dive angle, or other factors when performing the imaging observation activity. In addition, the common high-altitude airship is augmented by the efficient hybrid energy system, so as to enable it to be operated in long endurance [29]. However, this paper mainly focuses on the daily-scheduling problem with shorter task period. Therefore, the influence of the energy factors on the reconnaissance activities is put aside in the scheduling model.

**Definition 1**

If task_*i*_ and task_*j*_ are assigned to the same airship, and task_*j*_ is arranged to be executed just next to task_*i*_, then task_*i*_ is called the preceding task of task_*j*_, and task_*j*_ is the following task of task_*i*_.

Given a decision matrix *X* = [*x*_*i*,*k*_]_*n*×*m*_, if task_*i*_ is arranged to be executed on *a*_*k*_, then *x*_*i*,*k*_ equals “1”; otherwise, *x*_*i*,*k*_ is “0”. If *x*_*i*,*k*_ *x*_*j*,*k*_ = 1, and task_*i*_ is the preceding task of task_*j*_, then *y*_*i*,*j*_^*k*^ is “1”; otherwise, let *y*_*i*,*j*_^*k*^ equals “0”.

In this paper, the main constraints are considered as follows.

*Constraint 1*. The airships execute observation activity within the task period, and each task only can be executed within its time window. 

*Constraint 2*. If a task is executed, then the execution time of this task is no less than the required duration. 

*Constraint 3*. Each task only can be assigned to one observation resource and just need to be done once. 

*Constraint 4*. Only one preceding task and one following task of each task are allowable at most. 

*Constraint 5*. The preemptive service in the task execution is prohibited. Once the execution starts, the process cannot be terminated until completion.

*Constraint 6*. Before the airship observes a new task, enough time should be given to change the observation position. 

*Constraint 7*. The airship observes a task must meet its lowest imaging resolution.

*Constraint 8*. The airship only cruises among different tasks;

*Constraint 9*. With regard to any two tasks which are executed by the same observation resource, the execution should be in sequence.

Assume TB_*k*_ is the task benefit obtained by *a*_*k*_ and RD_*k*_ is its cruising distance. The primary optimization objective of the collaborative scheduling problem for the high-altitude airships is to maximize the task benefit:

(1) $$\begin{array}{c} \underset{}{\max}\;z_{1}\left(X\right)=\sum_{k=1}^{m}{\text{TB}}_{k}. \end{array}$$

The calculation method of TB_*k*_ is

(2) $$\begin{array}{c} {\text{TB}}_{k}=\sum_{{\text{task}}_{i}\in \text{Task}}x_{i,k}p_{i}. \end{array}$$

On the basis of ensuring this objective, it is essential to shorten the cruising distance as far as possible.

(3) $$\begin{array}{c} \underset{}{\min}z_{2}\left(X\right)=\sum_{k=1}^{m}{\text{RD}}_{k}. \end{array}$$

If *x*_*i*,*k*_ = 1 and ∑_task_*j*_∈*Q*_ *y*_*j*,*i*_^*k*^ = 0, it means that task_*i*_ is the first task to be executed on *a*_*k*_; then, save the serial number of task_*i*_ in a variable *F*_*k*_. Furthermore, with any two points *A* = (*x*_*i*_, *y*_*i*_) and *B* = (*x*_*j*_, *y*_*j*_), we record the distance between them as *d*(*A*, *B*) = ||*A*−*B*||_2_. The calculation method of RD_*k*_ is expressed as follows:

(4) $$\begin{array}{c} {\text{RD}}_{k}=\underset{{\text{task}}_{i},{\text{task}}_{j}\in \text{Task}}{\sum \sum }y_{i,j}^{k}d\left({\text{sit}}_{i},{\text{sit}}_{j}\right)+d\left(o_{k},{\text{sit}}_{F_{k}}\right). \end{array}$$

Assume *R*_0_ is the resolution space of *X*. Let *R*_1_ be the optimal solution set while *z*_1_(*X*) is optimized separately. Establish the constraint-programming model of the collaborative scheduling problem for high-altitude airships as follows:

(5) z1(X∗)=max⁡  X∈R0z1(X),z2(X∗)=min⁡X∈R1  z2(X),s.t.  {[tbi,tei]⊂[tai,tdi]⊂[tstart,tend]tei−tbi≥ti, if∑k=1mxi,k=1∑k=1mxi,k≤1∑i=1nyi,jk≤1,  ∑j=1nyi,jk≤1[tbi,tei]∩[tbj,tej]=∅, if  xi,k·xj,k=1tei+di,jspeedk−K(1−xi,jk)≤tbjui≥RPjxi,j∑k=1myi,ik=0yi,jk·yj,ik=0xi,k,yi,jk∈{0,1}∀i,j∈{1,2,…,m},  k∈{1,2,…,n},

where the former nine inequalities correspond to the aforementioned nine constraints, respectively, and the tenth inequality restricts the span of the decision variables.

## 3. Cooperative Scheduling Method

### 3.1. Designing of Scheduling Frame

There exist numerous constraints in the collaborative scheduling problem of the high-altitude airships, so it is hard to solve this problem directly. 

**Figure 2:**
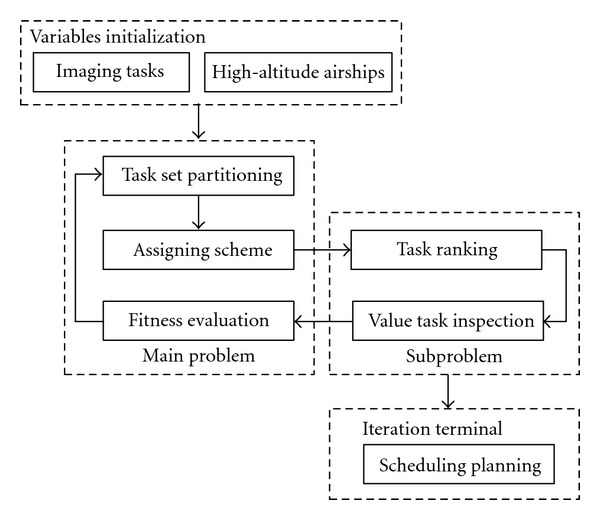
Architecture of cooperative scheduling algorithm.

As depicted in Figure 2, the hierarchical architecture is presented to convert the original problem into a main problem (namely, the task set partitioning) and a subproblem (namely, the cruising path selection).

**(1) The Main Problem**

The task set is divided into *m* portions according to the number of the observation resources. Each portion is called a cluster and denotes the assigning task set of an airship. The mapping relation between tasks and airships is constructed for decreasing the solution space of the original problem and facilitating the follow-up selection of cruising path.

**Definition 2**

Let *S*_*k*_ = {task_*k*1_, task_*k*2_, …, task_*kq*_*k*__} be the cluster corresponding to *a*_*k*_ and regard the cluster set *S* = {*S*_1_, *S*_2_, …, *S*_*m*_} as a feasible solution of the main problem, if the following conditions are satisfied:

(6) $$\begin{array}{c} \underset{S_{i}\in S}{\cup }S_{i}=S,\;\forall S_{i},S_{j}\in S,\;\;i\neq j,\;\;\text{if}\;S_{i}\cap S_{j}=\emptyset . \end{array}$$

Obviously, the task set partitioning problem belongs to the combinatorial optimization problem, and the scale of the solution space exponentially increases with the number of tasks and observation resources. At present, the global optimal solution algorithm with polynomial time complexity is nonexistent. Thus, this paper proposes a rapid algorithm named K-NGCA to solute this problem.

**(2) The SubProblem**

The priority for executing the sequence of each task is determined based on the solution of the main problem. The value tasks are selected according to the matching results between the observation capacity of airships and the requirement of tasks. In other words, the key nodes that each airship needs to fly through in sequence during the cruising are selected.

**Definition 3**

For any candidate task task_*i*_ on *a*_*k*_, if it can not be executed in its deadline, then task_*i*_ is regarded as an invalid task on *a*_*k*_; otherwise, task_*i*_ is a value task on *a*_*k*_.

The sub-problem mainly includes two aspects: one is to determine the priority execution sequence of each task (called the task ranking problem), which can be achieved by PA algorithm; the other is to select value task which should be observed (called the value task inspection problem), and it can be realized by KNSA algorithm.

### 3.2. K-NGCA Algorithm

**Definition 4**

Let *σ*_*k*_ = (*cx*_*k*_, *cy*_*k*_) be the geometric position center point of the tasks in the cluster *S*_*k*_ = {task_*k*1_, task_*k*2_, …, task_*kq*_*k*__}. *σ*_*k*_ is called the core of *S*_*k*_, and it can be calculated as follows:

(7) $$\begin{array}{c} cx_{k}=\frac{1}{\dim||s_{k}||}\sum_{{\text{task}}_{ki}\in S_{k}}tx_{ki},\\ cy_{k}=\frac{1}{\dim||s_{k}||}\sum_{{\text{task}}_{ki}\in S_{k}}ty_{ki}, \end{array}$$

where the operator dim⁡||·|| is used to calculate the element number of a set.

**Definition 5**

The nearness degree *r*_*i*,*k*_ denotes the relative distance between task_*i*_ and *S*_*k*_, and it can be calculated as follows:

(8) $$\begin{array}{c} r_{ik}=\frac{\sqrt{{\left(tx_{i}-cx_{k}\right)}^{2}+{\left(ty_{i}-cy_{k}\right)}^{2}}}{\sum_{j=1}^{m}\sqrt{{\left(tx_{i}-cx_{j}\right)}^{2}+{\left(ty_{i}-cy_{j}\right)}^{2}}}. \end{array}$$

In the initial construction of the cluster set, it is essential to avoid excessive elements which are assigned to a single cluster. This is beneficial to achieve the load balancing of the observation resources. Therefore, it is feasible to set a threshold variable Num to restrict the element number in a single cluster, which can be defined as

(9) $$\begin{array}{c} \text{Num}=\underset{a_{i}\in A}{\max}\left\{\text{ceil}\left(\frac{{\text{speed}}_{i}}{\sum_{a_{i}\in A}^{}{\text{speed}}_{i}}m\right)\right\}, \end{array}$$

where ceil (·) is a rounding function to be used to convert a decimal to an integer.

According to the position layout of the high-altitude airship, K-NGCA uses Greedy algorithm [30] to achieve the initiation of the cluster set. The main steps are shown as follows.

**Step 1**

All tasks in set task are sorted by their value indexes in descending order. The sequence is saved in set *S*Task.

**Step 2**

The cluster set *S* = {*S*_1_, *S*_2_,…, *S*_*m*_} is constructed, where *S*_*i*_ ← NULL, *σ*_*i*_ ← *o*_*i*_, *i* = 1,2,…, *m*.

**Step 3**

For each task task_*i*_ in set  *S*Task, let the set *F*_*i*_ = {*a*_*i*1_, *a*_*i*2_, …, *a*_*ie*_} contain the airships which meet the imaging resolution constraint of task_*i*_. Then, the subset *S*′ = {*S*_*i*1_, *S*_*i*2_, …, *S*_*ie*_} of set *S* is constructed.

**Step 4**

The nearness degrees between task_*i*_ and each element in *S*′ are calculated by (8), which are presented as *R*_*i*_ = {*r*_*i*1_, *r*_*i*2_, …, *r*_*ie*_}.

**Step 5**

Let *r*_*ik*_ = min⁡_*r*_*ij*_∈*R*_*i*__⁡{*r*_*ij*_}, and remove task_*i*_ from *S*Task into *S*_*ik*_.

**Step 6**

If *S*Task ≠ *∅*, then proceed to Step 3; otherwise, the algorithm ends.

K-NGCA adjusts the cluster scheme by iteration method. The pseudocode of K-NGCA is outlined as Algorithm 1.

In the pseudocode of K-NGCA, the cluster set is initiated by Greedy (see line 1). If the number of iterations is less than the threshold value MaxNum, then the cluster is repartitioned again. Otherwise, K-NGCA ends (see line 3). In the iteration process, each cluster is set as null firstly. The element number of each cluster is stored in vector *Q* (see line 6). According to the executing priority sequence *S*Task, each task task_*i*_ is assigned in sequence (see lines 8–31). The observation resources which meet the lowest resolution of task_*i*_ are selected and the corresponding clusters are saved. Search the cluster which has element number no more than Num and has the minimum nearness degree to task_*i*_; then, task_*i*_ is add into this cluster (see lines 11–23). If such cluster is nonexistent, then find out the observation resources which meet the lowest resolution of task_*i*_, but there is no restriction to the element number of the corresponding cluster. Select the cluster which has the minimum nearness degree to task_*i*_ and add task_*i*_ into this cluster (see lines 25–27). If such resource cannot be found, then task_*i*_ is rejected (see line 29). Update the cores of all clusters after iteration (see line 33).

### 3.3. PA Algorithm

The task ranking is a combination optimization issue, which is commonly solved by the intelligence algorithm (IA). The traditional IA involves simple operation and fast convergence, but it also has certain faults, such as inaccuracy search and early maturing. For instance, the classic PSO algorithm tends to terminate evolution in the iteration, because the particles tend to quickly gather to the historically optimized position of swarm. Similarly, while a superior chromosome is presented in the traditional GA algorithm, its genetic information may be rapidly spread to the swarm and make the algorithm being fast trapped into the local optimal solution. In this paper, a novel IA with the ability to avoid this prematurity is proposed.

PA is an evolutionary computation technique based on the propagation process of biological flock. The main principle of PA is summarized from animal swarm behavior, which indicates that individuals struggle to survive by competition and cooperation. In this algorithm, the survival activities of the flock are abstracted into three kinds of simple events, that is, search for food, breeding new individuals, and death. Similar to other swarm intelligent algorithms, each member in a flock is an independent individual, and its basic features include the search position and age. PA uses the iteration to implement the survival process and employs the minimum age unit of the population as iteration step. Each iteration is called a propagation and the aforementioned simple events are included. In order to find adequate food, all the individuals determine their new search positions in the next propagation based on their personal experience as well as information gained through inheritance or interaction. This process is accompanied with the feeble individual death and the new individual birth. Death means that several individuals are eliminated, and only the survivors can participate in the next propagation. The causes of individual death include

1. inadequate food is found out due to the bad search position, so that the individuals die of hunger;
2. the long survival time induces individuals to get aged, and elder individuals to die with the natural life gradually;
3. the accidental death which is caused by other factors, for example, prey, disease, and accident.

It is essential to choose the individuals who are suitable to breed new members to participate in propagation. This operation is important to maintain the population scale and deliver the search position information of the excellent individuals. The main steps of PA are summarized as follows.

**Step 1**

The swarm is initialized with random search position and survival age of all individuals.

**Step 2**

Each search position is evaluated by fitness function, and the global optimal search positions of the individual and the population are stored, respectively.

**Step 3**

The hunger and the aging degree of each individual are analyzed, and then we can judge the dead ones in the swarm.

**Step 4**

If the propagation criterion is met, then go to Step 5; otherwise, go to Step 6.

**Step 5**

The suitable individuals are selected as parents to breed and initialize the child individuals.

**Step 6**

Move to the new search position and update the individual age.

**Step 7**

If the termination criterion is satisfied, then the iteration ends; otherwise, go to Step 2.

The aforementioned steps are the basic concepts of PA. There still needs to clear the encoding rule, search method, and propagation mechanism of this algorithm.

#### 3.3.1. Encoding Rule

Decimal encoding rule is applied to represent search position of the individual with a Multidimensional vector, called the position vector, where the elements in the vector correspond to the task number. In the decoding operation, sort all tasks according to the value of the corresponding elements in a descending order, then the position vector can be converted into the task priority sequence.

For example, the task set *S*_1_ = {task_1_, task_2_, …, task_6_} is assigned to airship *a*_1_. In encoding operation, the search position of each individual is denoted by six-dimensional vector. Assume [0.27, 0.54, 0.95, 0.63, 0.15, 0.91] is a position vector, which denotes the execution priority sequence of task_5_, task_1_, task_2_, task_4_, task_6_, and task_3_. If this position vector is changed to [0.96, 0.48, 0.81, 0.14, 0.37, 0.92], then the execution priority sequence is altered to task_4_, task_5_, task_2_, task_3_, task_6_, and task_1_.

Additionally, the twotuples AI_*i*_ = 〈*F*_*i*_, *G*_*i*_〉 are employed to denote fitness of individual in swarm, where *F*_*i*_ is the task benefit and *G*_*i*_ is the cruising distance of *i*th individual, respectively. The comparison method of fitness is given as follows:

(10) $$\begin{array}{c} {\text{AI}}_{1}>{\text{AI}}_{2},\;\;\text{if}\;F_{1}>F_{2},\\ {\text{AI}}_{1}>{\text{AI}}_{2},\;\text{if}\;F_{1}=F_{2},\;\;G_{1}<G_{2},\\ {\text{AI}}_{1}={\text{AI}}_{2},\;\text{if}\;F_{1}=F_{2},\;\;G_{1}=G_{2},\\ {\text{AI}}_{1}<{\text{AI}}_{2},\;\text{otherwise}. \end{array}$$

#### 3.3.2. Search Method

In the process of searching a new position, the individuals are more inclined to move towards the global optimal search position where the population has appeared. However, there exists a certain distance between the current search position of the individuals and the global optimal search position of the swarm, so the individuals have to move many times to arrive at the destination. But the individuals may enter into the worse search position result in the deviation of movement direction. In order to reduce this risk, it is essential for the individual to consider the relationship among its own global optimal search position and current search position and the global optimal search position of the swarm in each movement.

Assume the population scale of algorithm is *N*. In the *t*th propagation, the global optimal position vector of the swarm is *GZ* ^*t*^ = (*gz*_1_^*t*^, *gz*_2_^*t*^,…, *gz*_*q*_*i*__^*t*^); the current and the optimal position vectors of individual agent_*i*_ are *Z*_*i*_^*t*^ = (*z*_*i*,1_^*t*^, *z*_*i*,2_^*t*^,…, *z*_*i*,*q*_*i*__^*t*^) and *BZ*_*i*_^*t*^ = (*bz*_*i*,1_^*t*^, *bz*_*i*,2_^*t*^,…, *bz*_*i*,*q*_*i*__^*t*^). If agent_*i*_ can survive in the *t* + 1th propagation, *Z*_*i*_^*t*+1^ can be calculated according to the following equations:

(11) $$\begin{array}{c} v_{i,j}^{t}=z_{i,j}^{t}+R_{1}\left(bz_{i,j}^{t}-z_{i,j}^{t}\right)+R_{2}\left(gz_{j}^{t}-z_{i,j}^{t}\right),\\ z_{i,j}^{t+1}=v_{i,j}^{t}+\Delta w_{i,j}^{t},\;i\in \left[1,N\right],\;\;j\in \left[1,q_{i}\right], \end{array}$$

where *v*_*i*,*j*_^*t*^ is the *j*th element on the undisturbed position vector *V*_*i*_^*t*^ in *t*th propagation, *R*_1_, *R*_2_ ∈ (0,1) is the random variable, and Δ*w*_*i*,*j*_^*t*^ ∈ (0,1) is the random disturbance variable of *v*_*i*,*j*_^*t*^.

#### 3.3.3. Propagation Mechanism

In the iteration process, there are individuals which die of hunger, old, and accident reasons. The scale of population is set as a constant *N* in order to maintain the stable search ability of algorithm. If the individual number *M* is less than *N*, then select *N* − *M* members whose survival time span is [Age_1_, Age_2_] and food is adequate as the parents are to breed new individuals, so as to supplement swarm. If the number of suitable individuals is less than *N* − *M*, then take all individuals as parents, and the absent ones can be generated based on the random method (it can be regarded to acquire the absent parents externally). This operation that the new individuals are generated by the parents can refer to the choosing operation and the crossover operation in genetic algorithm (GA) [21].

In *t*th iteration, assume the aging degree of agent_*i*_ is OD_*i*_(*t*) and the hunger degree is HP_*i*_(*t*). The probability of accidental death due to other reasons is expressed as the constant *a* ∈ (0,1), and the survival possibility of agent_*i*_ after the *t*th propagation is given as follows:

(12) $$\begin{array}{c} {\text{LP}}_{i}\left(t\right)=\left(1-a\right)\left[1-{\text{OD}}_{i}\left(t\right)\right]\left[1-{\text{HP}}_{i}\left(t\right)\right]. \end{array}$$

Calculate the survival possibility of all individuals in the swarm and determine the death by the roulette after normalization. The quantitative methods of aging degree and hunger degree are introduced as follows.

**(1) Aging Degree**

Usually, the search process of the traditional swarm intelligent algorithm easily drags into the local optimization. The main reason is that when the individuals with the dominant fitness appear in the swarm, their characteristic information will spread to other individuals rapidly and promote the swarm to converge towards the dominant individuals. In order to avoid the shortages of orthodox swarm intelligent algorithm, PA records the aging degree of each individual in every propagation and controls the expected survival time of each individual, so as to adjust the convergence speed of the algorithm.

Assume age_*i*_(*t*) is the age of agent_*i*_ in the *t*th propagation. If agent_*i*_ survives after the *t* + 1th propagation, let age_*i*_(*t* + 1) = age_*i*_(*t*) + 1; otherwise, let age_*i*_(*t* + 1) = 1. The aging degree of agent_*i*_ in *t*th propagation is defined as

(13) $$\begin{array}{c} {\text{OD}}_{i}\left(t\right)=1-\exp\left(\frac{-{\text{age}}_{i}\left(t\right)}{E\text{Age}}\right), \end{array}$$

where *E*Age is the expected survival time of each individual.

In particular, if age_*i*_(*t*) = 0 and OD_*i*_(*t*) = 0, it shows that agent_*i*_ is in a completely young state and that the death possibility of aging is nonexistent. If age_*i*_(*t*) → *∞* and OD_*i*_(*t*) = 1, it shows that agent_*i*_ will be in a continuous aging state with the increasing propagation time and that the death possibility infinitely approaches to 1.

**(2) Hunger Degree**

It is essential to reevaluate the hunger degree of each individual after propagation in order to analyze the performance of searching position. Generally speaking, the global optimal solution of the problem which we research cannot be obtained in advance, so the relative optimization is viewed as the criterion to measure the search position of the individuals. All individuals are sorted according to their fitness in a descending order after *t*th propagation, and the sequence is stored as Fit_*t*_ = {Fit_*t*1_, Fit_*t*2_,…, Fit_*tN*_}. Meanwhile, the first *h* individuals in Fit_*t*_ are assumed in hunger. The hunger degree of the individuals is given as follows:

(14) $$\begin{array}{c} {\text{HP}}_{ki}\left(t\right)=\{\begin{array}{c} \varepsilon ,\;\text{if}\;i\leq h,\\ 0,\;\text{if}\;i>h, \end{array} \end{array}$$

where *ε* ∈ (0,1) is the death probability cause of hunger.

#### 3.3.4. Parameters Analysis

The main parameters of PA include the population size, iteration number, hunger individual number, starvation probability, expected survival age, breeding age, and accidental probability. We will analyze these parameters in the following, and this work focuses on the parameter selection of PA.

**(1) Population Size**

The searching ability of PA is inadequate when the population size is small, so the iteration number should be increased to acquire a better solution. On the contrary, a large population size is conducive to a strong searching ability, but it could also lead to a low convergence speed. However, PA has the ability to jump the local optimal solution regardlessofpopulation size.

**(2) Iteration Number**

The iteration number is usually a constant, which is employed to adjust the searching time of algorithm. PA may not be able to find the ideal optimized solution with a small iteration number. If this parameter is enlarged, it is possible for PA to achieve a low searching efficiency as the searching time is increased.

**(3) Hunger Individual Number and Starvation Probability**

The product of hunger individual number and starvation probability is regarded as the expected number of dead one in a single iteration. The elimination rate of the inferior individuals is fast when this expected number is large this will help to improve the searching quality of swarm, but it will also incur a low convergence speed. On the contrary, a smaller value generates a faster convergence speed of PA because the swarm tends to keep the existing individuals, but prematurity is inevitable.

**(4) Expected Survival Age**

The expected survival age is proposed to control the convergence speed of swarm. The random searching of PA is presented in the solution space when this value is small. But a large value will also be invalid to acquire an ideal solution due to the rapid convergence of PA.

**(5) Breeding Age**

The position information of superior individuals is allowed to be spread to warm, which is similar to the genetic operation of GA. The difference is that PA controls the spreading velocity of the position information about the superior individuals by setting the breeding age. This parameter is assigned in the latter part of survival age because individuals are common in their best searching position at this time interval. Meanwhile, the little survival time of superior individuals prevents its position information from being spread excessively.

**(6) Accidental Probability**

The accidental probability is used to simulate the emergency death of swarm's evolution, which can decide the mutation probability of individual. Adaptive mutation can help the algorithm to jump the local optimal solution, but the extensive mutation will destroy the stable search of swarm in the solution space.

### 3.4. KNSA Algorithm

According to the priority execution sequence of tasks acquired by PA, the key nodes are determined that each airship needs to fly through at a given constant speed and altitude. The cruising path optimization method between the successive key nodes can be learned from [24, 31, 32]. 

**Figure 3:**
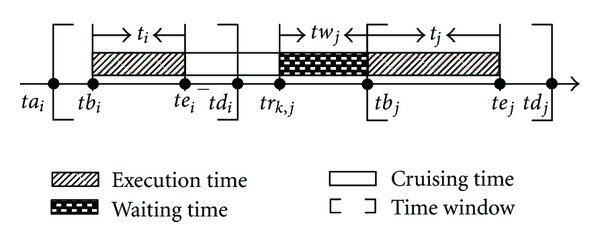
The waiting time between the successive key nodes.

For any task, it can only be executed within its time window. If we can observe task_*i*_ before allowable execution timing instant *ta*_*i*_, then the waiting time should be introduced as is presented in Figure 3.

Figure 3 shows that task_*i*_ and task_*j*_ are two neighboring tasks on *a*_*k*_; *tr*_*k*,*j*_ is the preparation time of *a*_*k*_ before task_*j*_ is executed; *tw*_*k*,*j*_ is the waiting time of *a*_*k*_ before observing task_*j*_. *tr*_*k*,*j*_ and *tw*_*k*,*j*_ can be calculated as follows:

(15) $$\begin{array}{c} {tr}_{k,j}={tb}_{i}+t_{i}+d\left({\text{sit}}_{i},{\text{sit}}_{j}\right){\text{speed}}_{k}^{-1},\\ {tw}_{k,j}=\max\left\{{tb}_{j}-{tr}_{k,j},0\right\}. \end{array}$$

With regard to the waiting time for an airship, it can be solved in many ways. For instance, the airship waits firstly and then reaches the location of the next task at the stipulated cruising speed or reaches location of the next task firstly and observes this task until the allowable moment. 

The executing timing interval of task_*i*_ can be easily determined as follows:

(16) $$\begin{array}{c} {tb}_{j}={tr}_{k,j}+{tw}_{k,j},\\ {te}_{j}={tb}_{j}+t_{j}. \end{array}$$

Due to the limited maneuverability of the airship, some tasks may not be executed before their deadlines. Therefore, the execution value of a task decreases with the time advancement, and this trend is irreversible.

Assume *S* = {*S*_1_, *S*_2_,…, *S*_*m*_} is a partitioning solution of the task set, where *S*_*k*_ is the task set assigned to *a*_*k*_, and the elements in *S*_*k*_ have been sorted by the task priority in sequence. The method of detecting the value tasks and designing its execution timing interval is shown in Algorithm 2.

 In Algorithm 2, the value tasks detection and the execution timing interval assignment are synchronized. The task sets assigned to various observation resources are scheduled by the algorithm, respectively. The variables including the preparation time, the task benefit, and the cruising path of each observation resources are initialized (see lines 2-3). According to the task priority execution sequence, the execution value of each task in set *S*_*i*_ is detected in order (see lines 3-4). If task_*i*_ can be executed in its time window, then task_*i*_ is a value task. The algorithm calculates the execution time of task_*i*_ and removes it to the key node set *G*_*k*_ (see lines 5–8); otherwise, task_*j*_ is an invalid task which will not be arranged in the execution queue (see line 10). Set the decision variables by ranking result in *G*_*k*_ (see lines 13–18).

## 4. Experiment Designing

In this section, simulation experiment is conducted to illustrate the effectiveness of the proposed method. The proposed algorithms are implemented by Matlab 2007 on a laptop with Pentium IV 3.06 GHz CPU, 2 GB memory, and Windows XP operating system. As far as we know, there are no accepted benchmarks yet in cooperative scheduling problem of high-altitude airships, so the random models are used to construct the application scenario and simulate the battlefield area with 200 × 300 km^2^.

### 4.1. Simulation Setup

Three high-altitude airships are tested in the experiment, and the task period is 0*∼*24 h. The main parameters for high-altitude airships simulation are listed in Table 1.

The task number varies from 50 to 300 as six instances, and they are deployed randomly within the battlefield. The task value indexes are changed from 1 to 10, and the imaging resolution is 0.3*∼*0.6 m. The equation of generating the time window for tasks is presented as follows:

(17) $$\begin{array}{c} {td}_{i}={ta}_{i}+\left(1+T\text{Base}\right)\times t_{i}, \end{array}$$

where *T*Base is used to adjust the tightness of the time window.

The setting of task parameters offers the flexibility to simulate the various workloads for high-altitude airships.  Table 2 gives the configuration of task parameters employed in our experiment. We check the performance impact of parameters Task number, *T*Base, and Task length by using the “once tuning one parameter (OTOP)” experiment method, which can be found in many researches [33–35]. The evaluation objects include task benefit and cruising distance.

In order to evaluate the effectiveness of K-NGCA algorithm, the Greedy algorithm which has been mentioned in the former section is applied in the task set partitioning. Furthermore, we verify the effectiveness of PA by comparing it with GA and PSO. We incorporate those algorithms to yield four new algorithms named K-NGCAPA, GreedyPA, K-NGCAGA, and K-NGCAPSO, respectively. The small-scale experiments have been finished to obtain the adaptive parameters of the PA, which are listed in Table 3.

### 4.2. Performance Impact of Task Number

**Figure 4:**
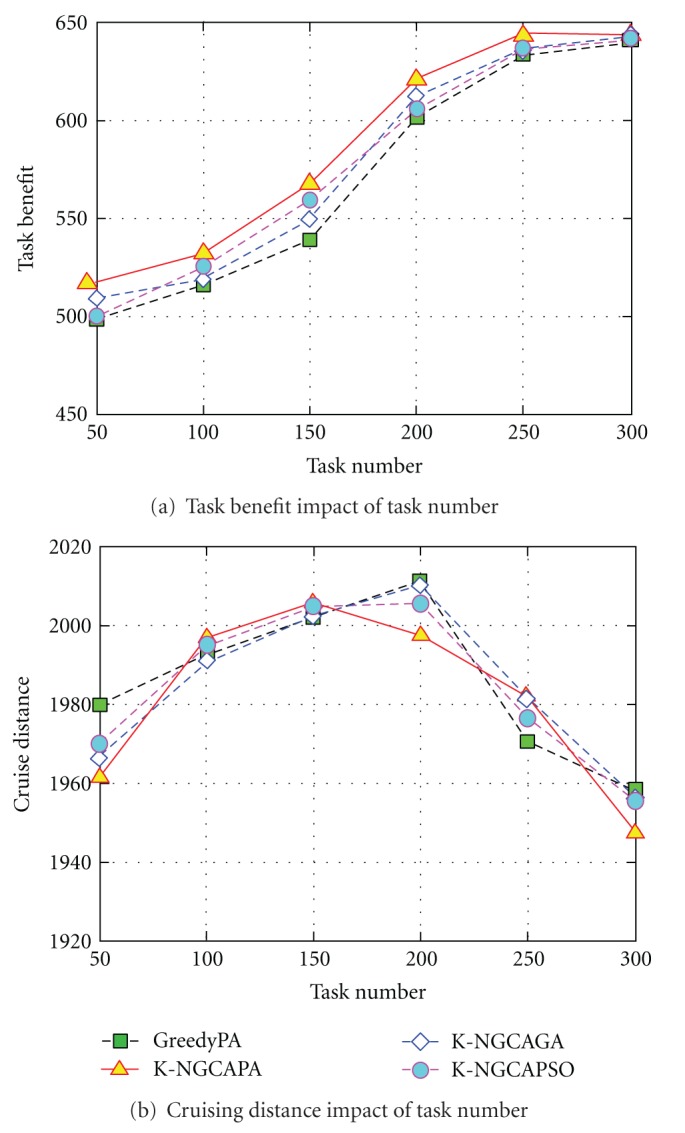
Scheduling results in various task number.

In this experiment, we investigate the impact of task number on the performance of these algorithms.  Figure 4 plots the scheduling results.

Figure 4(a) shows that K-NGCAPA obtains a higher task benefit than other algorithms (GreedyPA, K-NGCAGA, or K-NGCAPSO). In various task scales, the task benefit obtained by K-NGCAPA can be higher than that of GreedyPA, and K-NGCAGA, K-NGCAPSO reaching 0.384%, 2.92%, and 2.71%, respectively, which shows a very high scheduling performance. 

From Figure 4(a), we can observe that the task benefit increases gradually with the task number, but this tends to be flat while the variable is up to more than 200. This ascribes that the scale of value tasks increases gradually thus brings about the increasing number of the executable tasks and conduces to gain more task benefit. However, due to the limited observation capacity of the airships, when the task number ascends to a certain extent, the task benefit tends to increase slowly. It can be seen from Figure 4(b) that the cruising distance of the airships increases firstly and then decreases while the task number becomes larger. This cause of increasing task number will bring about the higher task density in the battlefield. In case the task density is low, a longer cruising distance needs to be flied if the airship is assigned to execute more tasks. While the task density reaches to a certain degree, the airships can execute more tasks just in a shorter scope. Therefore, the cruising distance declines.

### 4.3. Performance Impact of Time Window

**Figure 5:**
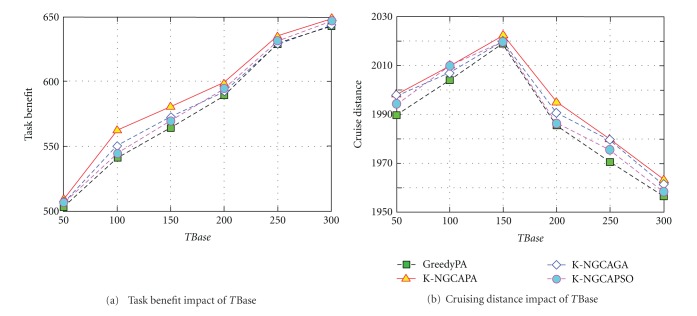
Scheduling results in various *T*Base.

The objective of this experiment is to investigate the performance impact of time window on the task guarantee ability. We divide *T*Base into six levels from 1 to 6.  Figure 5 depicts the different scheduling results in various *T*Base levels.

The experimental results in Figure 5(a) show that the K-NGCAPA achieve higher guarantee ratios than GreedyPA, K-NGCAGA, and K-NGCAPSO. It can be seen that the task benefit increases with extending *T*Base. This ascribes that the increase of *T*Base brings about a loose time window. In particular, the task deadline is extended, so more tasks can be executed timely. Hence, the task benefit increases gradually. From Figure 5(b), it can be seen that the cruising distance increases firstly and then decreases with the enlarging *T*Base. In the initial stage, the more tasks that airships execute are at the cost of longer cruising distance. Therefore, the cruising distance increases firstly. However, when the time window becomes loose, the tasks which are within the shorter distance range of the airships but once are unable to be executed timely become executable. Thus, the execution priority will be given to such tasks, and the scheduling scheme is adjusted by the new priority sequence which brings about the decline of the cruising distance.

### 4.4. Performance Impact of Task Length

**Figure 6:**
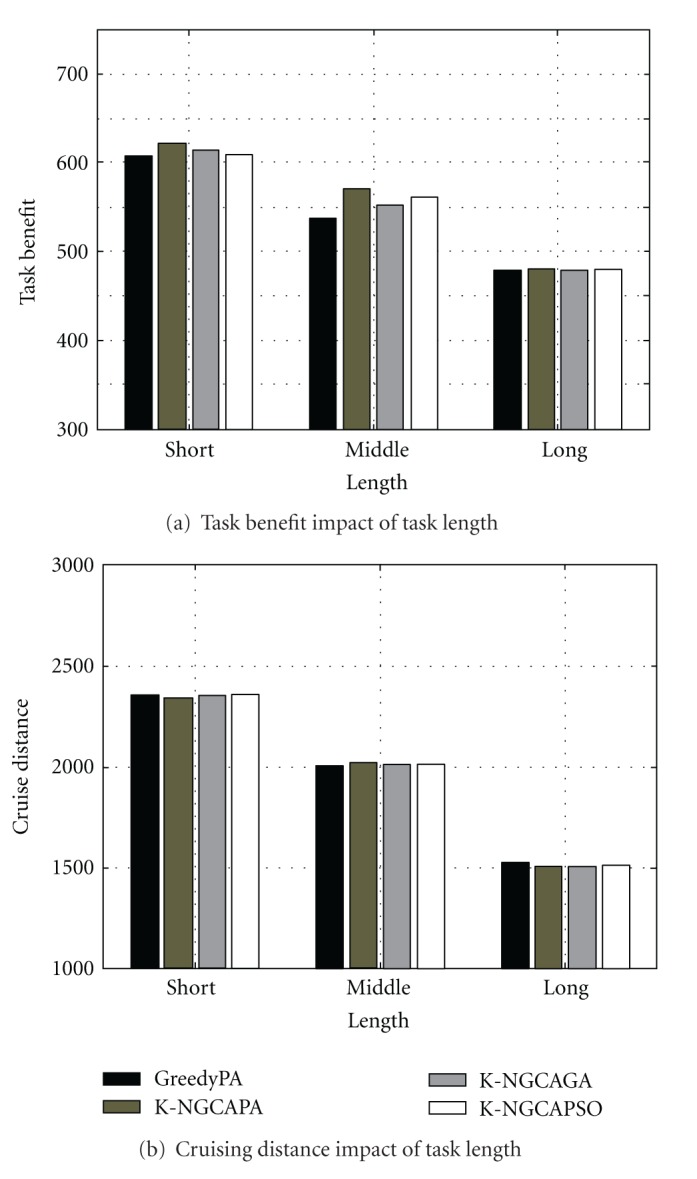
Scheduling results in different task length.

To examine the performance impact of task length, three test configurations of task length can be found in Table 2. We assume the task length is satisfied with the uniform distribution.  Figure 6 plots the scheduling results under short, middle, and long tasks.

From Figure 6(a), it can be seen that the task benefit decreases gradually when the task length is extended. This is because that the required executing time of the single task become long, so that the tasks with later sequence cannot be executed timely. At the same time, the cruising distance is shortened with the decreasing of executable tasks. This trend can be observed from Figure 6(b).

Again, we can observe from Figures 4 and 6 that the effects of the task number and time window on the optimization objects are positive, that is, both the increase of task number or extension of time window will enlarge the task benefits. Meanwhile, the effect of task length on the optimization objects is negative. Then, we can find the relationship between the aforementioned factors. In the given application scenarios, the task benefits are maintained by enlarging the time window or reducing the task length if the task number is reduced. On the contrary, while the time window is reduced or the task length is increased, we could also maintain the task benefits by increasing the task number.

## 5. Conclusions and Future Work

The cooperative scheduling of imaging observation tasks for high-altitude airships are a kind of complex combinatorial optimization problem. This paper makes the following contributions in the study of this problem.

1. The main constraints of the cooperative scheduling problem for high-latitude airships are presented. The timeliness of imaging observation tasks is proposed, and the influence of this feature on the reconnaissance activities is summarized. On the basis of the above analysis, we construct the constraint programming model.
2. A hierarchical solution frame is developed to solve this cooperative scheduling problem. The original scheduling problem is converted into a main problem (task set partitioning) and a sub-problem (cruising path selection). It is available to simplify the solution process.
3. The K-NGCA algorithm is proposed to partition the task set based on the position relationship between the airships and tasks. The simulation results proved the effectiveness of this algorithm.
4. A new swarm intelligent algorithm is presented, which is called PA algorithm. This algorithm can control the population convergence speed by adjusting the expected survival time of the individuals.
5. The generation method of the application scenario for high-altitude airships is provided, and the influences of the parameters (such as the task number, the task length, and the time window) on the reconnaissance capability of high-altitude airships are analyzed by experiment.

Also for our future work, we plan to analyze the influences of the parameters (such as the expected survival time, the breeding age, and the accidental probability, etc.) on the convergence speed of PA algorithm. This work plays an important role in avoiding the defects that the traditional swarm intelligent algorithms are fast to fall into the local optimal solution effectively.

## Figures and Tables

**Algorithm 1:**
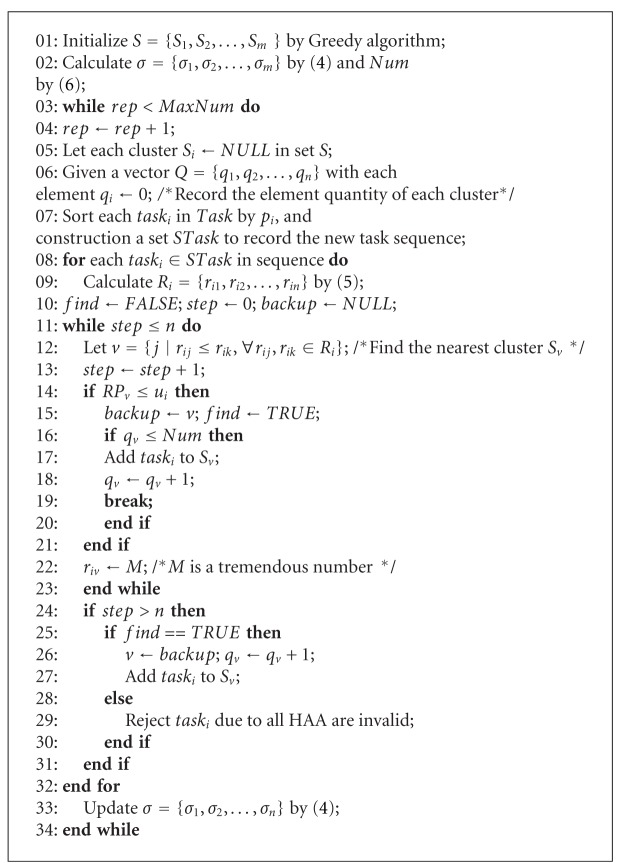
Pseudocodes of K-NGCA algorithm.

**Algorithm 2:**
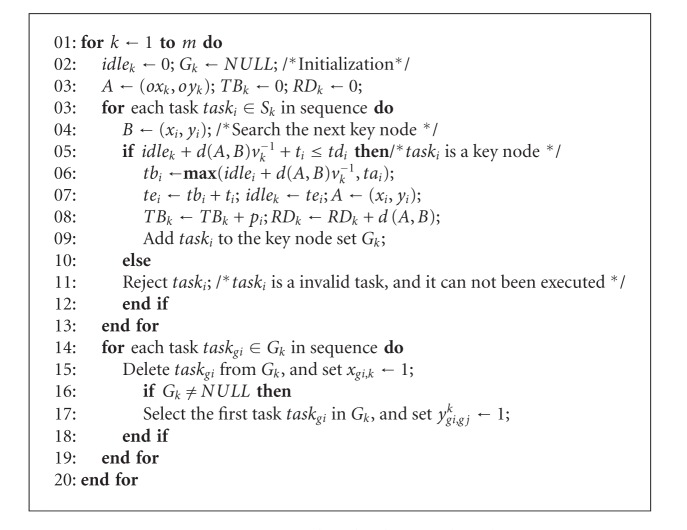
Pseudocode of KNSA algorithm.

**Table 1:** Parameters for high-altitude airships simulation.

HAA	Initial position	Speed (km/h)	Imaging resolution (m)
*a*_1_	(20, 30)	60	0.61
*a*_2_	(150, 170)	75	0.36
*a*_2_	(280, 30)	70	0.24

**Table 2:** Parameters for task simulation.

Parameters	Value (fixed)−(varied)
Task number	(200)−(50, 100, 150, 200, 250, 300)
Value index	([1, 10])
Imaging resolution (m)	([0.3, 0.6])
TBase	(10)−(5, 10, 15, 20, 25, 30)
Task length (min)	([15, 30)−([0, 15], [15, 30], [30, 45])

**Table 3:** Parameters for PA algorithm.

Parameters	Value (fixed)
Population size	30
Iteration number	200
Hunger individual number	5
Breeding age	[5, 30]
Expected survival age	35
Accidental probability	0.05
Starvation probability	0.1
